# Getting to Fidelity: Consensus Development Process to Identify Core Activities of Implementation Facilitation

**DOI:** 10.1007/s43477-024-00119-5

**Published:** 2024-04-18

**Authors:** Jeffrey L. Smith, Mona J. Ritchie, Bo Kim, Christopher J. Miller, Matthew J. Chinman, P. Adam Kelly, Sara J. Landes, JoAnn E. Kirchner

**Affiliations:** 1 VA Behavioral Health Quality Enhancement Research Initiative (QUERI) & HSR&D Center for Mental Healthcare & Outcomes Research (CeMHOR), Central Arkansas Veterans Healthcare System, 900 S. Shackelford Road, Fifth Floor, Little Rock, AR 72211, USA; 2 Department of Psychiatry, University of Arkansas for Medical Sciences, 4301 W. Markham Street, #755, Little Rock, AR 72205, USA; 3 VA Behavioral Health QUERI & HSR&D Center for Healthcare Organization and Implementation Research (CHOIR), VA Boston Healthcare System, Boston, MA 02130, USA; 4 Department of Psychiatry, Harvard Medical School, Boston, MA 02115, USA; 5 VA Pittsburgh Healthcare System, Research Office Building (151R), University Drive C, Pittsburgh, PA 15240, USA; 6 Southeast Louisiana Veterans Healthcare System, 2400 Canal Street (11F), New Orleans, LA 70119, USA

**Keywords:** Implementation facilitation, Fidelity, Implementation strategy, Modified Delphi consensus development process

## Abstract

Transferring successful implementation strategies from research to practice requires approaches for assessing fidelity to the strategy’s core components. Implementation facilitation (IF) is a strategy involving an interactive process of problem-solving, enabling, and supporting individuals in efforts to implement clinical innovations that occurs in the context of a recognized need for improvement and supportive interpersonal relationships. Because IF is a dynamic strategy involving numerous activities, our objective was to conduct a rigorous consensus development process to identify core activities for monitoring fidelity to IF when applied in clinical settings. We first conducted a scoping literature review to identify the range of activities used when IF has been applied in clinical settings, searching multiple citation databases for English-language articles including “facilitation” or other commonly-used terms for the strategy published from 1996–2015. Through multi-stage screening, 135 articles (from 94 studies) were identified for data extraction on IF activities, frequency with which IF activities were identified as ‘core’ by study authors, and study outcomes. From the literature review, we identified 32 distinct IF activities and developed definitions/examples for each. Next, we conducted a 3-stage, modified-Delphi expert panel consensus development process to identify core IF activities across three implementation phases (i.e., Pre-Implementation, Implementation, Sustainment). The expert panel identified 8 core activities for the Pre-Implementation Phase, 8 core activities for the Implementation Phase, and 4 core activities for the Sustainment Phase. This work provides an important foundation for developing measures/tools to assess use of core IF activities to ensure the strategy is delivered with fidelity.

Within the field of implementation research, the concept of ‘fidelity’ generally applies to two different, but related, areas: fidelity to the *clinical innovation*, and fidelity to the *implementation strategy* (Shelton et al., 2023). In this paper, *clinical innovation* refers to evidence-based clinical practices or any clinical practice, program, or initiative to be implemented (Ritchie et al., 2020a). *Implementation strategy* refers to methods used to enhance the adoption, implementation, sustainment, and scale-up of a clinical innovation (Kirchner et al., 2023; Proctor et al., 2013). *Fidelity* has been defined as the extent to which core elements of either a clinical innovation or an implementation strategy are delivered as intended (Kirchner et al., 2023; Shelton et al., 2023). This paper focuses on a comprehensive body of work undertaken to identify core activities that may be used to assess fidelity to one strategy, implementation facilitation (IF) (Harvey & Kitson, 2015; Powell et al., 2015; Ritchie et al., 2020a, frequently used to support implementation of clinical innovations.

To ensure appropriate transfer of successful implementation strategies from research to practice, it is essential to first identify the strategy’s core components (Michie et al., 2009) so the strategy can be applied effectively (i.e., with fidelity) not only within the context of research studies but also in other non-research clinical quality improvement efforts (Kirchner et al., 2023; Shelton et al., 2023). This is important for ensuring that the strategy retains its potency and impact in terms of supporting implementation of clinical innovations with fidelity so anticipated improvements in clinical care and outcomes are realized. Such transfer from research to practice is a primary goal of novel training programs designed to disseminate effective implementation strategies outside of research settings and increase practitioners’ knowledge and skills in applying them, for example, the U.S. Veterans Health Administration Quality Enhancement Research Initiative’s Implementation Strategy ‘Learning Hubs’ (Goodrich et al., 2020; Kirchner et al., 2022). Unfortunately, in comparison to developing measures to ensure fidelity to effective clinical innovations, researchers have given relatively little attention to developing measures to help ensure fidelity to successful *implementation strategies* (Slaughter et al., 2015), although some have begun to address this gap by identifying core elements for monitoring fidelity to implementation strategies such as ‘evidence-based quality improvement’ (Stockdale et al., 2020) and ‘technical assistance’ (Dunst et al., 2019).

Implementation facilitation (IF) is a dynamic strategy involving an interactive process of problem-solving, enabling, and supporting stakeholders in their efforts to adopt and incorporate clinical innovations into routine practice that occurs in the context of a recognized need for improvement and supportive interpersonal relationships (Perry et al., 2019; Powell et al., 2015). Typically, IF bundles an integrated set of activities that together support implementation of effective practices (Ritchie et al., 2023). Examples of such IF activities include engaging stakeholders, action planning, problem-solving, providing technical support, and tailoring implementation to local context without compromising fidelity to the clinical innovation (Perry et al., 2019; Stetler et al., 2006). The specific activities used in an IF strategy may vary from setting to setting depending on a number of factors, such as organizational readiness (e.g., culture, climate), infrastructure, available resources, leadership support, organizational goals/priorities, and motivation of stakeholders (Damschroder et al., 2022a, 2022b; Harvey & Kitson, 2016; Ritchie et al., 2023). This variability in activities that may be applied in an IF strategy makes it particularly challenging but important to determine whether there may be a set of ‘core’ activities that should be more commonly applied across settings and which could be used to assess and ensure fidelity to the strategy.

A growing number of studies have shown IF to be an evidence-based strategy (Kirchner et al., 2022) for implementing complex evidence-based practices and other clinical innovations (Kilbourne et al., 2014; Kirchner et al., 2014; Nutting et al., 2009; Ritchie et al., 2017; Wang et al., 2018). For example, in U.S. Department of Veterans Affairs (VA) clinical settings, IF has been used successfully to: implement primary care mental health integration (PCMHI) programs with improved uptake, quality, and adherence to evidence (Kirchner et al., 2014; Ritchie et al., 2017); improve uptake of a national program to re-engage veterans with serious mental illness into care (Kilbourne et al., 2015); improve adoption of brief cognitive behavioral therapy in primary care (Mignogna et al., 2014); improve metabolic side effect monitoring for patients taking antipsychotic medications (Kirchner et al., 2016); and increase enrollment in an initiative to improve transitions of care for patients with heart failure (Heidenreich et al., 2015). In non-VA clinical settings, IF has been used to support implementation of patient-centered medical homes in primary care (Nutting et al., 2009) and improve diabetes care (Dickinson et al., 2014), preventive care (Baskerville et al., 2001), and pediatric hospital care (Ayieko et al., 2011). Further, one systematic review found that primary care practices were almost three times more likely to adopt evidence-based guidelines through the use of IF (Baskerville et al., 2012), while another review found beneficial effects of IF on outcomes of four major chronic diseases in primary care: asthma, cancer, cardiovascular disease, and diabetes (Wang et al., 2018). Clearly, evidence for the impact of IF in supporting implementation of effective clinical practices is robust across diverse clinical settings, including under-resourced, late-adopter locations (Kirchner et al., 2014). However, despite ample evidence for the impact of IF in implementing clinical innovations, some studies have shown that IF may not produce anticipated results when the strategy is not delivered as intended (Rycroft-Malone et al., 2018; Seers et al., 2018). Accordingly, it is vital to identify core IF activities that may be used for monitoring fidelity to the IF strategy to increase the likelihood for implementation success.

This paper describes methods and results from a comprehensive body of work to first inform and then execute a rigorous, multi-stage expert panel consensus development process to identify core activities that may be used to assess IF fidelity when the strategy is applied in clinical settings. This work is foundational for developing measures/tools to assess use of core IF activities to ensure the strategy is delivered with fidelity and to help ensure successful transfer of the strategy from research to implementation practice.

## Methods

### Scoping Literature Review

We conducted a scoping review to analyze and synthesize a broad collection of literature on IF studies from multiple disciplines to identify and define the range of activities used when IF has been applied in clinical settings. Scoping reviews are useful for mapping and clarifying key concepts underpinning a research area as well as clarifying working definitions and/or the conceptual boundaries of a topic (Arksey & O’Malley, 2005). These reviews involve synthesis and analysis of a wide range of research to provide greater conceptual clarity about a specific topic (Davis et al., 2009) and may be particularly relevant to disciplines with emerging evidence (Levac et al., 2010). We elected to conduct a scoping review because, as noted above, IF is dynamic in that it typically involves a collection of integrated activities to support implementation of clinical innovations. Further, the literature pertaining to IF is broad and diffuse in that: (a) investigators have used a variety of different labels and terminology to describe IF; (b) the strategy has been applied across diverse clinical settings; (c) investigators have used a number of different frameworks to guide the application and evaluation of IF (e.g., integrated—Promoting Action on Research Implementation in Health Services (i-PARIHS) (Harvey & Kitson, 2015), Consolidated Framework for Implementation Research (CFIR) (Damschroder et al., 2022a, 2022b), Replicating Effective Programs (REP) (Kilbourne et al., 2013)); and (d) specific activities reported in the literature vary from study to study when IF has been applied in clinical settings. Although seminal work in the nursing literature has proposed taxonomies of facilitation activities (Dogherty et al., 2010; Elnitsky et al., 2015), sub-stantive operational definitions for activities included in these taxonomies have not been provided and the broader field of implementation scientists/practitioners have not yet embraced any single taxonomy.

To inform our expert panel consensus development process, the scoping review focused on answering the following questions: (1) What distinct activities have been used when IF has been applied in clinical settings, and how can these activities be defined?; (2) How frequently have the activities been reported as ‘core’ or ‘critical’ to the strategy when applied?; (3) Among studies reporting outcomes, how frequently have the IF activities been used in studies reporting positive implementation, process-of-care, and/or patient outcomes?

**Fig. 1:**
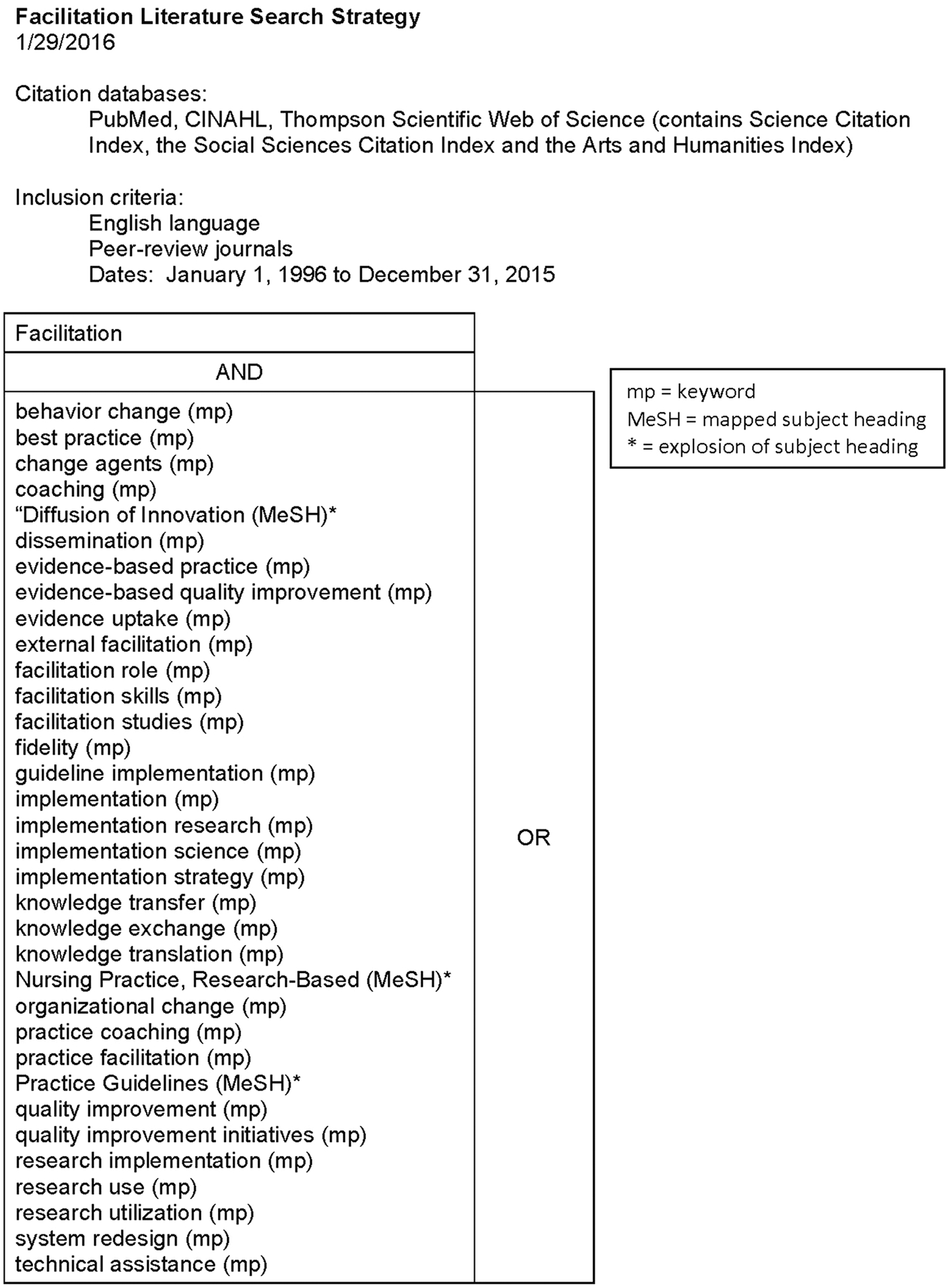
Search Strategy for Scoping Literature Review

Consistent with scoping review guidelines (Arksey & O’Malley, 2005; Levac et al., 2010; Peters et al., 2020), we conducted a multi-step review process. In addition to identifying our questions of interest, our process included development and application of a search strategy, selection of studies, extraction of data, refinement of a previously developed code list to identify facilitation activities in the literature, further revision of this list for use in our consensus development process, and summation of additional findings from the scoping review. The search strategy was informed by review articles and key studies of IF and applied in PubMed, Cumulative Index of Nursing and Allied Health Literature (CINAHL), and Thompson Scientific Web of Science citation databases. On January 29, 2016, we searched for English-language articles that included the term “facilitation” and other terms related to the strategy (e.g., practice facilitation, coaching, change agents) published from January 1996 – December 2015 (see Fig. 1). It is important to note that – in related work to develop an updated IF literature collection for public access (Ritchie et al., 2022b) – review of 24 additional IF studies published from 2016–2020 (selected at random) identified *>no* additional IF activities beyond those used to inform our expert panel consensus development process described below. *This indicates that our scoping review of IF studies from 1996–2015 to inform expert panelists’ decisions on core IF activities was robust and still relevant currently. Accordingly, for transparency and clarity, this paper provides the scoping review results that expert panelists actually considered and used in their deliberations to identify core activities for monitoring fidelity to the IF strategy*.

**Fig. 2:**
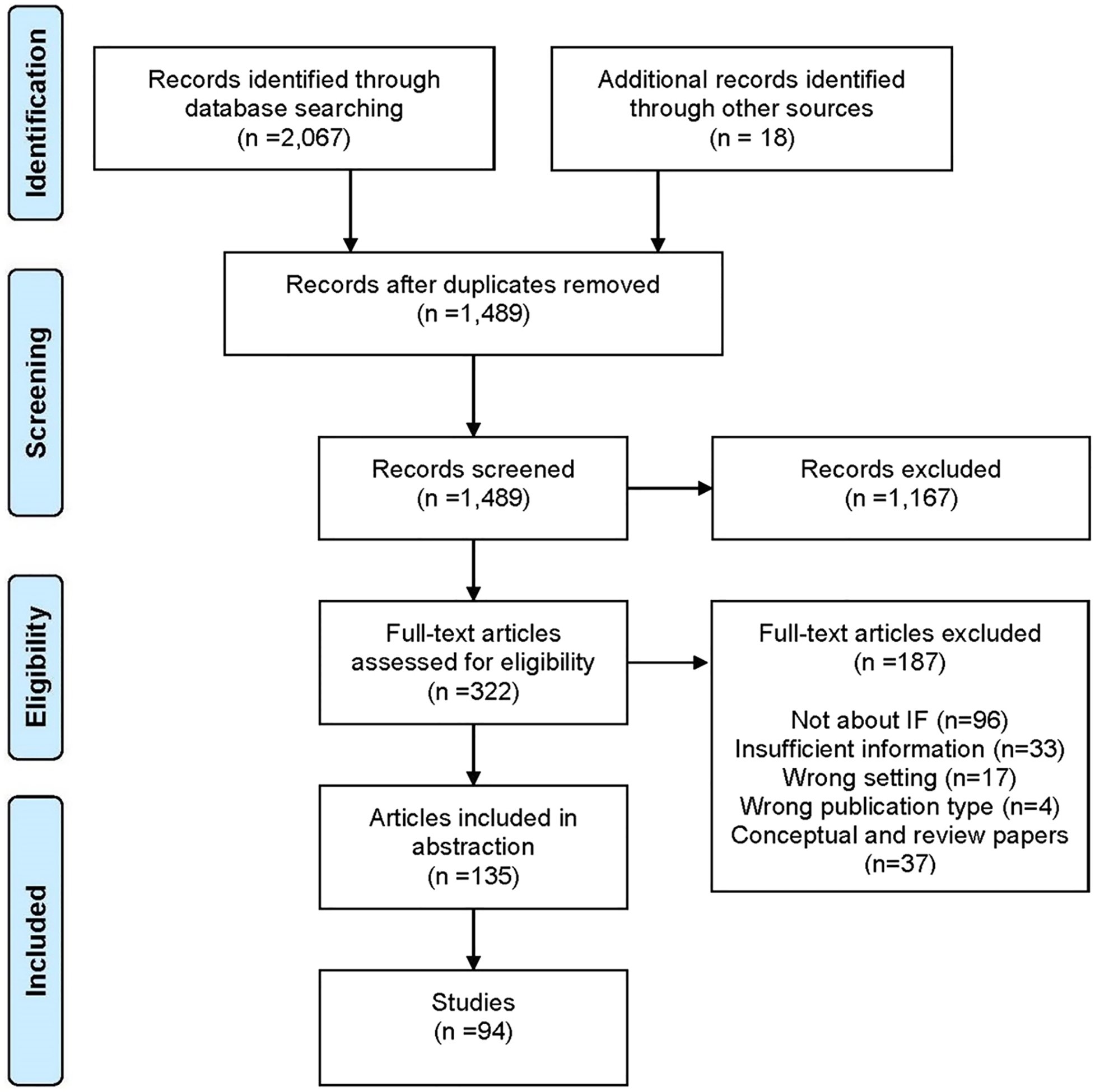
PRISMA Chart for Scoping Literature Review

Our literature search identified 1471 citations and abstracts, which were imported into Covidence systematic review software (“Covidence systematic review software,”) for screening and full-text review. Two implementation facilitation experts (JLS and MJR) conducted first-stage screening to assess relevance, with 322 articles identified through mutual agreement by the two reviewers for second-stage full-text review. Ten percent of the full-text articles were reviewed by both reviewers for inter-rater reliability testing, with the reviewers showing a high level of agreement (96.8% agreement; Cohen’s kappa 0.93) on decisions regarding whether the article should be included/excluded for data extraction. The remaining articles were divided between the two reviewers to complete the full-text review. During the full-text review process, eighteen additional articles were identified and included. We only included articles from studies conducted in clinical settings, and we did not include conceptual articles or commentaries as our objective was to identify the breadth of IF activities that were actually applied in implementation research studies. Also, as is typical of scoping reviews (Levac et al., 2010; O’Brien et al., 2016), we did not conduct a quality appraisal of included studies. Ultimately, a total of 135 articles from 94 studies were selected for data extraction (see PRISMA chart in Fig. 2). Consistent with scoping review methodology (Arksey & O’Malley, 2005; Tricco et al., 2018), we included a broad array of articles in the final set including articles describing experimental or observational studies, use of quantitative and/or qualitative methods, and protocols from completed or ongoing studies.

For each study included, data were extracted to document activities used by facilitators (including whether authors identified specific activities as ‘core’, ‘critical’, or other synonymous terms to the success of the strategy) and any reported ‘implementation’, ‘process-of-care’, and ‘patient’ outcomes. For our purposes, positive ‘implementation’ outcomes referred to completion of specific tasks or development of specific resources/tools to support implementation of a clinical innovation, program, or practice (e.g., developing an action plan or implementation blueprint, establishing a patient registry, conducting a training, developing a clinical reminder, developing patient education materials, etc.). ‘Process-of-care’ outcomes refer to actions or interventions performed during the delivery of care (e.g., changes in medication prescribing, side effect monitoring, number of psychotherapy sessions, foot exams for people with diabetes, etc.). ‘Patient’ outcomes refer to the impact of a healthcare service or intervention on patient well-being (e.g., changes in symptoms, functioning, lab values, etc.). For process-of-care and patient outcomes, study reports of statistically significant improvements in either or both of these were determined to be positive outcomes.

Implementation facilitators conduct a wide variety of activities (Dogherty et al., 2010; Ritchie et al., 2023); and descriptions of IF activities in the literature we reviewed were highly variable. To assess the use of activities across studies, we extracted data of interest to create a summary for each study and adapted and updated a list of IF activities and operational definitions developed in a previous study (Ritchie et al., 2023) to create a code list. We then used Atlas.ti, version 7, to conduct qualitative analysis of the IF activities data extracted from the 94 studies. JLS initially coded all study summaries; MJR reviewed the initial coding and provided feedback; JLS reviewed the feedback; and then the two coders discussed discrepancies and reached consensus on final coding decisions. We then summarized the scoping review findings and further revised the names of the IF activities and their definitions to inform our expert panel consensus development process.

### Expert Panel Consensus Development Process

To identify core IF activities informed by the scoping review findings, we conducted a rigorous 3-stage modified Delphi consensus development process (Dalkey, 1969; Dalkey & Helmer, 1963; Linstone & Turoff, 2002) with a panel of 15 experts who had substantial experience in the application and/or evaluation of IF in clinical settings. We elected to use a modified Delphi process because it: (a) is effective for determining expert consensus when there is little or no definitive evidence and opinion is important; and (b) allows for independent ratings by experts in multiple rounds, followed by a final meeting of the panelists for interactive group discussion and collective decision-making to arrive at a consensus (Eubank et al., 2016; Pyne et al., 2020). The expert panelists were recruited from a learning community of over 150 implementation science researchers and practitioners focused on sharing best practices and developing new tools/resources to advance the science and practice of IF; i.e., the ‘Implementation Facilitation Learning Collaborative’ (Kirchner et al., 2022). The 3-stage expert panel process described below was conducted over a 9-month period in 2018–2019.

Since the complexity of clinical innovations can vary greatly and may influence panelists’ selection of core IF activities, panelists were asked to identify core activities separately for both a relatively high and a relatively low complexity clinical innovation example. The ‘high’ complexity clinical innovation example was implementation of measurement-based care for depression in primary care mental health integration (PCMHI) settings. The ‘low’ complexity clinical innovation example was implementation of *baseline* side effect monitoring for patients started on a new antipsychotic medication (see Online Resource 1 for more detailed descriptions of the high and low complexity innovation examples that were shared with panelists). For each of the innovations, panelists were asked to identify core IF activities for each of three phases of implementation (i.e., ‘Pre-Implementation’, ‘Implementation’ and ‘Sustainment’ phases (see Table 1)), as core IF activities were reasoned to likely differ for each of these phases (Kirchner et al., 2022). This 3-phase framework (Pre-Implementation, Implementation, Sustainment) for describing implementation activities has been used by other researchers as well (Kilbourne et al., 2019). For each phase of implementation, IF activities had to be selected by a super-majority of the expert panelists (≥ 60% threshold) to be included in the final set of core IF activities.

### Stage 1 of Expert Panel Process

The list of 32 IF activities and definitions identified in our scoping review were provided to panelists (see Online Resource 2). For both the ‘high’ and ‘low’ complexity clinical innovation examples, panelists were asked to select up to 10 core IF activities from this list for each phase of implementation that they felt were most critical for a facilitator to apply to support implementation of the clinical innovation. Panelists were allowed to select fewer than 10 core activities for a given phase if they wished but were not allowed to select more than 10 as our objective was to identify a limited set of core IF activities to monitor fidelity to the strategy while minimizing reporting burden on facilitators. Additional findings from the scoping review pertaining to implementation, process-of-care, and patient outcomes (see Table 2 below) were shared with the panelists to help inform their selections, though they were instructed to consider their own expertise and experience as well. In other words, the panelists were instructed to consider the IF activity definitions in Online Resource 2, data in Table 2, AND their own expertise/experience in making their selections, i.e., not to rely *exclusively* on one versus the others. Finally, for the core IF activities they selected, panelists were asked to distribute 100 points among them as an indicator of the relative importance of each activity from their perspective. Online Resource 3 provides a de-identified example of one panelist’s completed Stage 1 workbook for the high complexity clinical innovation example.

### Stage 2 of Expert Panel Process

Aggregate full-panel results from Stage 1 were shared with the 15 expert panelists, and each was given the option to change their Stage 1 core IF activity selections and/or importance point allocations if they wished. In Stage 2, 8 (53.3%) of the 15 panelists chose to make changes to their Stage 1 activity selections or importance point allocations; 7 (46.7%) elected to make no changes.

### Stage 3 of Expert Panel Process

Aggregate results from Stage 2 were shared with panelists in advance of an online group Skype meeting for discussion and final voting on core IF activities for each phase of implementation. Ten (67%) of the original 15 panelists participated in the online meeting. Stage 2 expert panel results showed a convergence of core IF activities selected for both the high and low complexity clinical innovation examples. Specifically, for both clinical innovations through Stage 2, 8 identical activities met the ≥ 60% super-majority threshold for the Pre-Implementation Phase, 5 identical activities met the ≥ 60% threshold for the Implementation Phase, and 3 identical activities met the ≥ 60% threshold for the Sustainment Phase. Thus, in Stage 3, panelists were not asked to differentiate selection of core IF activities between the two clinical innovation examples. IF activities meeting the ≥ 60% super-majority threshold for both the high and low complexity innovations in Stage 2 were automatically approved as core activities for Stage 3, while IF activities meeting the ≥ 60% threshold for one of the innovations but not the other in Stage 2 were presented sequentially to the panel for deliberation followed by a final vote (again using the ≥ 60% threshold) on whether the activity should be included in the final set of core IF activities.

## Results

### Scoping Literature Review Results

Based on review of the 94 studies, 32 distinct IF activities were identified and definitions/examples were developed for each (see Online Resource 2). Using a qualitative coding system developed in related work (Ritchie et al., 2022a, 2022b), the 32 IF activities are organized under 10 clusters (or categories) in Online Resource 2. Although the 32 IF activities are fairly well distributed across the 10 clusters, the four clusters with more IF activities than others were ‘Building relationships, teams, and networks’ (5 activities), ‘Enabling / fostering change’ (5 activities), ‘Planning/preparing for implementation’ (4 activities), and ‘Providing education/information’ (4 activities). The list of 32 IF activities and their definitions were provided to the expert panel to inform their decision-making on core IF activities.

Additional findings from the scoping review used to inform our expert panel consensus development process are provided in Table 2 above. The most frequently applied IF activities across the 94 studies were: conduct ongoing monitoring of program implementation (71%); clinical skills education (70%); engaging stakeholders, obtaining buy-in (67%); data collection to assess context and baseline performance (59%); providing updates and feedback (57%); and technical support (57%). Among 31 studies reporting ‘core’ IF activities, activities most frequently reported as core were: engaging stakeholders, obtaining buy-in (61%); data collection to assess context and baseline performance (48%); providing support (48%); conduct ongoing monitoring of program implementation (39%); and technical support (35%).

In studies reporting positive implementation outcomes (e.g., developing an action plan, developing a patient registry, providing training), Table 2 shows the most frequently used IF activities were: administrative tasks (80.0%); problem-solving (76.5%); adapting program to local context without compromising fidelity (74.1%); clinical skills education (74.0%); and managing group/team processes (70.0%). In studies reporting positive process-of-care (e.g., improvements in prescribing or lab monitoring) and/or patient outcomes (e.g., improvements in symptoms/functioning), the most frequently used IF activities were: administrative tasks (52.0%); data collection to assess context and baseline performance (51.3%); technical support (50.0%); fostering change (unspecified) (50.0%); clinical skills education (48.0%); and engaging stakeholders, obtaining buy-in (47.8%).

### Expert Panel Consensus Development Results

Table 3 provides overall results from the 3-stage, modified Delphi consensus development process engaged in by our expert panel to identify core IF activities for monitoring fidelity to the strategy for each of the three phases of implementation. At the conclusion of Stage 3, the expert panel had identified totals of 8 core activities for the Pre-Implementation Phase, 8 core activities for the Implementation Phase, and 4 core activities for the Sustainment Phase (see Table 3). Sixteen (50%) of the 32 IF activities identified in the scoping review were approved as a core activity for one or more of the three phases of implementation. As anticipated, the expert panel predominantly identified different core IF activities for the three phases of implementation, although four (25% of core activities) were identified as core for more than one phase.

Interestingly, 6 of the 8 core IF activities selected for the Pre-Implementation Phase by the expert panel were among the top 10 activities used in studies that showed positive implementation and/or process-of-care/patient outcomes (see Table 2); this was also the case for 7 of 8 core activities selected for the Implementation Phase, and 3 of 4 core activities selected for the Sustainment Phase. This indicates that while the expert panel may have given strong deference to findings from the scoping literature review to guide their collective decision-making, they also relied upon their own expertise and experience to make final selections of core activities, consistent with the instructions provided to them. One notable exclusion among core activities selected by the expert panel was ‘clinical skills education’, with some panelists making the case that such education is often provided by a subject matter expert (as a component of the clinical innovation itself) rather than the person serving in the IF role. Though there was a robust discussion among panelists on whether it should be a core activity, it ultimately did not meet the ≥ 60% super-majority threshold for any of the phases, reflecting our focus on identifying core activities for individuals serving in the IF role.

## Discussion

Based on a scoping literature review of 94 studies applying an implementation facilitation (IF) strategy in clinical settings, we identified 32 distinct IF activities. Building on previous work (Ritchie et al., 2023), we developed and refined operational definitions for each of the 32 activities. Then, through a rigorous modified-Delphi consensus development process (Dalkey, 1969; Dalkey & Helmer, 1963; Linstone & Turoff, 2002) informed by findings from the scoping review, an expert panel identified core activities that may be used for monitoring fidelity to the IF strategy for the three phases of implementation: 8 core activities for the Pre-Implementation Phase, 8 core activities for the Implementation Phase, and 4 core activities for the Sustainment Phase. The core IF activities for each phase are listed in Table 3, and definitions/examples for each are provided in Online Resource 2 (among the definitions/examples developed for all 32 IF activities identified in our scoping review). This work is foundational for developing measures/tools to assess use of core IF activities to ensure the strategy is delivered with fidelity (Shelton et al., 2023) and to help ensure successful transfer (or ‘hand-off’) of the strategy from research to implementation practice (Kirchner et al., 2022).

Going forward, facilitators’ use of core IF activities could be documented and tracked on adapted versions of IF Time Tracking Logs used in previous studies for systematically documenting facilitators’ time and activities for analyses, including cost (Miller et al., 2020; Ritchie et al., 2020a, 2020b) (generic versions of the IF Time Tracking Log with instructions for customization are available from the corresponding author on request). In an evaluation, one might conduct quantitative analyses of IF Time Tracking Log data to assess: (a) to what extent facilitators applied ALL of the core IF activities in the site(s) they worked with for each phase of implementation; (b) how frequently facilitators applied the different core IF activities in their contacts with sites (intensity); and (c) whether/how such IF fidelity measures were associated with study outcomes. In cases where facilitators in non-research clinical initiatives may not wish to document their time and activities on an IF Time Tracking Log (to minimize facilitator reporting burden), we have developed and are currently piloting stand-alone IF fidelity tool prototypes (quantitative surveys designed to periodically assess use of the core IF activities). Facilitators’ use of core IF activities may also be assessed in qualitative debriefing interviews by evaluators assessing their activities with sites to support implementation of a given clinical innovation (Ritchie et al., 2023). In sum, consistent inclusion of IF fidelity measures (based on assessment of the core IF activities identified in this work) in future studies may be used to examine and characterize empirical relationships between IF fidelity and study outcomes (e.g., clinical innovation uptake, and improvements in clinical care and patient outcomes) (Ritchie et al., 2020a). For example, rigorous ‘common elements’ approaches (Engell et al., 2023) may hold promise for synthesizing data on the use of multifaceted implementation strategies (like IF), within and across studies, and conducting tests of associations between discrete elements of these strategies (e.g., use of core IF activities) and study outcomes. These associations can be studied using causal inference (e.g., coincidence analysis (Whitaker et al., 2020)), case study research (e.g., matrixed multiple case study (Kim et al., 2020)), and other established approaches for examining common or heterogeneous individual elements and their combinations that relate to outcomes (e.g., methods to identify active psychotherapy elements (Leijten et al., 2021)). Such approaches may enhance our understanding of the active ingredients and mechanisms of change in complex implementation strategies like IF while taking into account specific contextual characteristics and other factors that influence successful implementation (Engell et al., 2023).

It is important to note that this study is *not* intended to suggest that facilitators should use *only* the core IF activities in their work to support implementation of clinical innovations (i.e., to the exclusion of using other non-core activities included among the full list of 32 activities when needed). That would be a complete misinterpretation of this work. As stated previously, IF is a dynamic strategy that gives facilitators the flexibility to apply different activities to support implementation of a given clinical innovation, taking into account characteristics of the innovation itself (e.g., complexity), the recipients (e.g., motivation to change), and the context of the settings in which the innovation is implemented (e.g., organizational readiness, available resources, leadership support) (Harvey & Kitson, 2015, 2016). To illustrate this point, 24 of the 32 IF activities were selected as a core activity in the Pre-Implementation Phase for the high complexity clinical innovation by at least one of the expert panelists through the first 2 stages of our expert panel process, yet ultimately only 8 activities met the ≥ 60% super-majority threshold for approval as a core activity in Stage 3. Given proliferation of the use of IF in research and clinical practice, our objective was to identify a *limited* set of commonly applied core IF activities (with operational definitions) that may be used for monitoring fidelity to the strategy without placing undue reporting burden on facilitators. Results indicate we were successful in meeting this objective in that while our consensus development process would have allowed as many as 10 IF activities to meet the ≥ 60% super-majority threshold to be identified as core for any given phase, ultimately no more than 8 core IF activities were identified for any one of the three phases. This suggests that our expert panelists were deliberative and selective in identifying core IF activities, consistent with our objective.

It may seem contradictory to identify a limited set of core activities for IF fidelity monitoring while also emphasizing that IF allows flexibility for activities to vary across settings depending on contextual and other factors. However, the findings from our consensus development process demonstrate that we were able to strike a balance between parsimony and flexibility. Eight of the 16 core IF activities identified by the expert panel relate to engaging stakeholders to gain a better understanding of their context, resources, and barriers and using that information to assist them with tailoring and adapting implementation of the clinical innovation to their setting (see Online Resource 2 definitions for ‘data collection to assess context and baseline performance’, ‘problem identification’, ‘problem-solving’, ‘engaging stakeholders / obtaining buy-in’, ‘action/implementation planning’, ‘adapting program to local context without compromising fidelity’, ‘fostering organizational change: structural’, and ‘conduct ongoing monitoring of program implementation’). Thus, these core activities are among those *fundamental* to maintaining fidelity to the IF strategy, as they can be used to identify the need for applying other (core and non-core) activities across diverse settings to help them be successful in their implementation efforts.

In related work, members of this research team also worked to identify ‘*Key Steps in Implementation Facilitation*’ (available at Implementation Facilitation Training Manual (v3) – Addendum 1 (va.gov)) consistent with those advocating for identifying core *functions* for fidelity assessment of complex health interventions (Perez Jolles et al., 2019). Again, because facilitators will typically use multiple activities in carrying out key steps in IF, and in response to requests from participants in our IF training program (Kirchner et al., 2022), we undertook the complementary work described herein to identify a limited set of more distinct core *activities* that can be used to assess fidelity to the IF strategy in implementation projects/initiatives.

### Strengths

Our literature review process is consistent with recommendations for enhancing scoping review methodology (Levac et al., 2010). Specifically, the review incorporated a clear purpose and research questions; applied an iterative team-based approach to selecting studies and extracting data; incorporated numerical summaries and qualitative thematic analysis for reporting of results; and incorporated intensive consultation with stakeholders (expert panel) in a rigorous consensus development process to consider the implications of review findings for research and practice as it pertains to monitoring fidelity to the IF strategy. The comprehensive and complementary approach to this scoping review and expert panel process may serve as a model for investigators who wish to address similar research questions to characterize the range of activities involved in other multifaceted implementation strategies and identify core activities that may be used for assessing fidelity to those strategies.

This work advances what has been learned from prior reviews of IF activities in several ways. First, our scoping review draws from studies conducted among multiple clinical disciplines, as opposed to those focusing on IF applied within a single discipline (e.g., nursing) (Dogherty et al., 2010; Elnitsky et al., 2015). Second, building upon prior work (Ritchie et al., 2023), this work further developed and refined operational definitions for the IF activities identified in our scoping review to try to provide greater clarity to the field. Further, in another scoping review, Cranley et al. (2017) identified nine facilitator roles and fifteen facilitation characteristics associated with use of research evidence at clinical sites. Our review complements and extends Cranley et al. by identifying and defining the full range of IF activities reported in the literature that may be associated with the facilitation roles and characteristics they identified. Finally, our expert panel process identified respective sets of core IF activities that may be used for monitoring fidelity to the strategy in the three different phases of implementation (i.e., Pre-Implementation, Implementation, and Sustainment phases), which is particularly important given that core activities were found to largely vary from phase to phase.

### Limitations

Despite the strengths of this work, there are potential limitations. First, identification of IF activities in the scoping review was dependent on which activities the study authors chose to report in publications. Thus, it is possible that: (a) some IF activities identified in the review may have been used more frequently across the 94 studies but were simply not reported in some cases and/or (b) some IF activities may have gone unreported entirely. Despite these possibilities, it is reasonable to consider that those IF activities that authors deemed most critical or core to their approach would have been reported. Second, scoping review results shared with the expert panelists only included articles published through 2015, which was necessary as our approach required: (a) screening, selecting, reviewing, and extracting data from relevant articles and studies; (b) qualitative coding and analysis of data; and (c) synthesizing findings from the scoping review so they could be used to inform the consensus development process to follow. After recruiting the expert panel, the multi-stage consensus development process itself took place over a 9-month period in 2018–2019, making it impractical to incorporate more recent publications at that point. However, as indicated in the Methods section, our review of an additional, randomly-selected 24 IF studies published from 2016–2020 did *not* identify any additional IF activities beyond those considered by our expert panel, indicating that our scoping review of IF studies published from 1996–2015 was robust and still relevant currently. Additionally, the core IF activities identified by the expert panel were subsequently shared with the broader membership of the Implementation Facilitation Learning Collaborative (Kirchner et al., 2022) for review/feedback, which resulted in no suggested changes to the expert panel’s decisions. Third, although expert panelists were able to identify core IF activities for each phase of implementation, it is not known whether implementation success may hinge upon the mere presence (at any level of frequency/intensity) of a given core activity in these phases, or instead, whether the frequency and/or intensity of use of the core IF activities may be more important. It may also be possible that certain combinations of core IF activities used together may have greater implications for implementation success (e.g., potential synergistic effects of core activities). These are important empirical questions to address in future research.

## Conclusions

Successful transfer of IF from research to practice requires measures that can be used to monitor and help ensure fidelity to core activities. Core IF activities for monitoring fidelity to the strategy were identified based on a comprehensive scoping review of the literature and a rigorous, multi-stage consensus development process. Further research is needed to evaluate the relationship(s) between IF fidelity measures (based on assessment of the core activities identified in this work), clinical innovation uptake, and improvements in clinical care and patient outcomes.

## Supplementary Material

Online Resource 3

Online Resource 2

Online Resource 1

## Figures and Tables

**Table 1:** Three Phases of Implementation

Pre-Implementation Phase:
This phase typically involves a facilitator’s initial engagement with site stakeholders over a period of weeks or months to gain a better understanding of their resources, priorities and context… with the primary objective during this phase being to assist them with developing an implementation plan
Implementation Phase:
Once the implementation plan has been developed, the ‘Implementation’ phase typically starts with a ‘kick-off’ where the implementation plan is carried out with ongoing relatively frequent contact by the facilitator to monitor and support the plan’s execution and refinement
Sustainment Phase:
During this phase, contact between site stakeholders is less frequent and eventually non-existent, with the objective being to turn a mature (hopefully successful) implementation effort over to the site with minimal further interaction with the facilitator

**Table 2:** Key Findings from Scoping Review of 94 Studies Applying an Implementation Facilitation Strategy

Implementation Facilitation (IF) Activity	# of studies reporting use of activity (from 94 studies)	% (rank, with top 5 bolded)	# of studies identifying activity as ‘core’ (from 31 studies reporting ‘core’ activities)	% (rank, with top 5 bolded)	# of studies reporting use of the activity and positive implementation outcomes^a^	% (rank, with top 5 bolded)^b^	# of studies reporting use of the activity and positive process-of-care and/or patient outcomes^a^	% (rank, with top 5 bolded)^b^
Conduct ongoing monitoring of program implementation	67	**71% (1)**	12	**39% (3)**	30/48	62.5 (13)	22/48	45.8 (10)
Clinical skills education	66	**70% (2)**	9	29% (6)	37/50	**74.0 (4)**	24/50	**48.0 (4)**
Engaging stakeholders, obtaining buy-in	63	**67% (3)**	19	**61% (1)**	31/46	67.4 (9)	22/46	**47.8 (5)**
Data collection to assess context and baseline performance	55	**59% (4)**	15	**48% (2)**	25/39	64.1 (11)	20/39	**51.3 (2)**
Providing updates and feedback	54	**57% (5)**	9	29% (6)	23/41	56.1 (14)	19/41	46.3 (9)
Technical support	54	**57% (5)**	11	**35% (4)**	27/40	67.5 (8)	20/40	**50.0 (3)**
Action / implementation planning	50	53% (6)	10	**32% (5)**	23/36	63.9 (12)	16/36	44.4 (11)
Providing support	49	52% (7)	15	**48% (2)**	26/38	68.4 (6)	18/38	47.4 (6)
Problem-solving	46	49% (8)	10	**32% (5)**	26/34	**76.5 (2)**	16/34	47.1 (7)
Adapting program to local context without compromising fidelity	41	44% (9)	7	23% (8)	20/27	**74.1 (3)**	9/27	33.3 (13)
Goal/priority setting	41	44% (9)	8	26% (7)	19/28	67.9 (7)	11/28	39.3 (12)
Managing group/team processes	40	43% (10)	8	26% (7)	21/30	**70.0 (5)**	14/30	46.7 (8)
Fostering change (unspecified)	39	41% (11)	4	13% (10)	17/26	65.4 (10)	13/26	**50.0 (3)**
Administrative tasks	34	36% (12)	9	29% (6)	20/25	**80.0 (1)**	13/25	**52.0 (1)**
Problem identification	33	35% (13)	7	23% (8)	17/23	73.9	13/23	56.5
Organizational change skills education	28	30% (14)	9	29% (6)	16/18	88.9	7/18	38.9
Fostering peer networking	26	28% (15)	4	13% (10)	17/21	81.0	9/21	42.9
Marketing	25	27% (16)	5	16% (9)	14/19	73.7	8/19	42.1
Fostering organizational change: structural	21	22% (17)	3	10% (11)	11/16	68.8	6/16	37.5
Developing shared vision / consensus building	17	18% (18)	5	16% (9)	8/10	80.0	2/10	20.0
Interceding and liaising with others	17	18% (18)	3	10% (11)	11/13	84.6	8/13	61.5
Identification / selection of local change agents	17	18% (18)	1	3% (13)	7/11	63.6	4/11	36.4
Describing / clarifying roles and responsibilities	15	16% (19)	3	10% (11)	7/12	58.3	7/12	58.3
Fostering networking with experts	14	15% (20)	4	13% (10)	6/8	75.0	2/8	25.0
Overcoming resistance to change	10	11% (21)	4	13% (10)	3/6	50.0	2/6	33.3
Fostering organizational change: cultural	9	10% (22)	0	–	4/4	100.0	1/4	25.0
Strategy/policy development	6	6% (23)	0	–	6/8	75.0	5/8	62.5
Pulling back and letting sites take lead	5	5% (24)	2	6% (12)	0/1	–	0/1	–
Attending, presenting at and/or organizing non-local meetings	3	3% (25)	0	–	3/3	100.0	0/3	–
Fostering spread of clinical innovation / facilitation methods	2	2% (26)	0	–	0/0	–	0/0	–
Helping to hire clinical program staff	2	2% (26)	0	–	2/2	100.0	1/2	50.0
Marketing education	1	1% (27)	0	–	0/1	–	0/1	–

a For these columns, denominators are the number of studies that reported use of the activity AND reported outcomes. Examples of positive ‘implementation outcomes’ include items such as developing an action plan or implementation blueprint, establishing a patient registry, conducting a training, implementing a clinical reminder, developing patient education materials, etc. Examples of positive ‘process of care outcomes’ include improvements in prescribing, side effect monitoring, number of psychotherapy sessions, foot exams for people with diabetes, etc. Examples of positive ‘patient outcomes’ include improvements in symptoms, functioning, lab values, etc

b For these columns, rankings are only provided for activities where denominator (number of studies) ≥ 25

**Table 3:** Core Implementation Facilitation (IF) Activities Identified by Expert Panel Members (*N* = 15)

	High Complexity Innovation		Low Complexity Innovatio	
Pre-Implementation Phase				
Core IF activities	N (%)	Mean ‘importance’ score (range)	N (%)	Mean ‘importance’ score (range)
Action / implementation planning^a^	15 (100%)	13.0 (5–30)	15 (100%)	13.3 (5–20)
Engaging stakeholders, obtaining buy-in^a^	14 (93%)	14.8 (10–20)	14 (93%)	14.4 (10–20)
Identification/selection of local change agents^a^	14 (93%)	12.3 (10–20)	11 (73%)	15.0 (10–30)
Data collection to assess context and baseline performance^a^	13 (87%)	12.2 (5–30)	14 (93%)	12.9 (8–30)
Describing/clarifying roles and responsibilities^a^	10 (67%)	7.3 (2–12)	12 (80%)	9.6 (5–15)
Administrative tasks^a^	10 (67%)	9.4 (5–15)	11 (73%)	7.7 (5–15)
Goal/priority setting^a^	10 (67%)	8.8 (5–11)	10 (67%)	9.1 (5–15)
Problem identification^a^	10 (67%)	9.5 (5–15)	10 (67%)	8.5 (5–10)
Implementation Phase				
Problem-solving^a^	13 (87%)	11.8 (5–15)	13 (87%)	11.5 (10–16)
Providing support^a^	13 (87%)	9.6 (4–15)	14 (93%)	10.5 (5–20)
Conduct ongoing monitoring of program implementation^a^	12 (80%)	10.3 (5–15)	13 (87%)	14.2 (10–20)
Fostering organizational change: structural^a^	10 (67%)	10.4 (9–15)	10 (67%)	11.9 (7–20)
Administrative tasks^a^	9 (60%)	9.1 (5–15)	9 (60%)	9.0 (5–20)
Managing group/team processes^b^ *(approved in 3*^*rd*^ *stage of expert panel)*	13 (87%)	12.1 (5–20)	8 (53%)	9.5 (5–15)
Providing updates and feedback^b^ *(approved in 3rd stage of expert panel)*	8 (53%)	10.1 (10–11)	13 (87%)	12.1 (5–20)
Adapting program to local context without compromising fidelity^b^ *(approved in 3*^*rd*^ *stage of expert panel)*	10 (67%)	8.6 (5–10)	5 (33%)	6.6 (3–10)
Sustainment Phase				
Pulling back and letting sites take lead^a^	14 (93%)	24.1 (10–40)	14 (93%)	28.6 (10–50)
Conduct ongoing monitoring of program implementation^a^	12 (80%)	12.9 (5–25)	13 (87%)	16.2 (10–25)
Providing updates and feedback^a^	9 (60%)	17.8 (10–50)	11 (73%)	17.3 (10–40)
Providing support^b^ *(approved in 3rd stage of expert panel)*	10 (67%)	10.3 (5–20)	8 (53%)	9.4 (5–20)

a Approved as a core IF activity for fidelity monitoring by meeting the ≥ 60% super-majority threshold through the first two stages of the expert panel process

b Approved as a core IF activity for fidelity monitoring in the 3rd stage of the expert panel process by meeting the ≥ 60% super-majority threshold on the final (Stage 3) panel vote. Implementation Phase: ‘Managing group/team processes’ (100% vote; 10/10); ‘Providing updates and feedback’ (60% vote; 6/10); and ‘Adapting program to local context without compromising fidelity’ (90% vote; 9/10). Sustainment Phase: Providing support (90% vote; 9/10)

## Abbreviations

CFIR: Consolidated Framework for Implementation Research
CINAHL: Cumulative Index of Nursing and Allied Health Literature
IF: Implementation facilitation
i-PARIHS: Integrated—Promoting Action on Research Implementation in Health Services
PCMHI: Primary care mental health integration
REP: Replicating Effective Programs
VA: U.S. Department of Veterans Affairs
