# Supplementary material for: Getting to Fidelity: Consensus Development Process to Identify Core Activities of Implementation Facilitation
Source: Glob Implement Res Appl. Author manuscript; Available in PMC 2024 May 17. (PMC11100021; doi:10.1007/s43477-024-00119-5)
Supplement: Online Resource 3 [file NIHMS1990339-supplement-Online_Resource_3.pdf]

**Online Resource 3.** Example of completed expert panelist stage 1 workbook

**HIGH COMPLEXITY INNOVATION:**
**"Implementing Measurement Based Care for Depression in Integrated Primary Care Mental Health Integration (PC-MHI) Settings"**

**INSTRUCTIONS:**
*** For each of the 3 phases of implementation, please place an "X" in the cells of up to 10 activities that you think are MOST CRITICAL for a facilitator to apply to support implementation of the clinical innovation. You may select fewer than 10 'core' activities for a phase if you wish, but do not select more than 10 activities for any phase.**

*** Next, for the activities you selected, distribute 100 points among them to indicate how important that activity is from your perspective compared to the others. Remember, the sum total of points you assign for each phase should = 100.**

| Implementation Facilitation Activities | PRE-IMPLEMENTATION PHASE | For each activity selected, assign points (sum of points should = 100) | IMPLEMENTATION PHASE | For each activity selected, assign points (sum of points should = 100) | SUSTAINMENT PHASE | For each activity selected, assign points (sum of points should = 100) |
|---|---|---|---|---|---|---|
| Action/implementation planning | X | 10 | | 0 | | 0 |
| Adapting program to local context without compromising fidelity | | 0 | X | 10 | | 0 |
| Administrative tasks | | 0 | | 0 | | 0 |
| Identification/selection of local change agents | X | 15 | | 0 | | 0 |
| Attending, presenting at and/or organizing non-local meetings | | 0 | | 0 | | 0 |
| Providing education on clinical skills | | 0 | | 0 | | 0 |
| Conducting ongoing monitoring of innovation implementation | | 0 | X | 10 | | 0 |
| Data collection to assess context and baseline performance | X | 10 | | 0 | | 0 |
| Describing/clarifying roles and responsibilities | X | 5 | | 0 | | 0 |
| Developing shared vision / consensus building | X | 10 | | 0 | | 0 |
| Fostering spread of clinical innovation / facilitation methods | | 0 | | 0 | | 0 |
| Engaging stakeholders, obtaining buy-in | X | 20 | | 0 | | 0 |
| Fostering change, unspecified | | 0 | | 0 | | 0 |
| Fostering networking with experts | | 0 | | 0 | | 0 |
| Fostering peer networking | | 0 | X | 5 | X | 10 |

| **Implementation Facilitation Activities** | **PRE-IMPLEMENTATION PHASE** | **For each activity selected, assign points (sum of points should = 100)** | **IMPLEMENTATION PHASE** | **For each activity selected, assign points (sum of points should = 100)** | **SUSTAINMENT PHASE** | **For each activity selected, assign points (sum of points should = 100)** |
|---|---|---|---|---|---|---|
| Fostering organizational change: culture & climate | | 0 | | 0 | | 0 |
| Fostering organizational change: structural | | 0 | X | 20 | | 0 |
| Setting goals / priorities | X | 10 | | 0 | | 0 |
| Helping to hire clinical program staff | | 0 | | 0 | | 0 |
| Interceding and liaising with others | | 0 | | 0 | | 0 |
| Managing group/team processes | | 0 | X | 5 | | 0 |
| Marketing | X | 5 | | 0 | | 0 |
| Providing education on marketing | | 0 | | 0 | | 0 |
| Providing education on organizational change | X | 5 | | 0 | | 0 |
| Overcoming resistance to change | | 0 | X | 15 | | 0 |
| Problem identification | X | 10 | | 0 | | 0 |
| Problem-solving | | 0 | X | 5 | | 0 |
| Providing support using interpersonal skills | | 0 | X | 10 | X | 10 |
| Providing updates and feedback | | 0 | X | 10 | X | 50 |
| Pulling back / transferring roles | | 0 | | 0 | X | 20 |
| Assisting with strategy/policy development | | 0 | X | 10 | | 0 |
| Technical support | | 0 | | 0 | X | 10 |
| | | **100** | | **100** | | **100** |
