# Supplementary material for: Getting to Fidelity: Consensus Development Process to Identify Core Activities of Implementation Facilitation
Source: Glob Implement Res Appl. Author manuscript; Available in PMC 2024 May 17. (PMC11100021; doi:10.1007/s43477-024-00119-5)
Supplement: Online Resource 2 [file NIHMS1990339-supplement-Online_Resource_2.pdf]

Online Resource 2. Implementation facilitation (IF) activity definitions/examples

IF Activities	IF Activity Definitions/Examples
Building relationships, teams, and networks	
Engaging stakeholders, obtaining buy-in	Engaging relevant stakeholders and seeking their participation/buy-in; building relationships with stakeholders; helping stakeholders 'own' the change.
Fostering networking with experts	Linking to external expert contacts; fostering contact with external people/organizations (non-peers) who can provide needed information or services.
Fostering peer networking	Fostering networking, communication across or within sites, learning collaboratives / communities of practice; cross-pollination using opinion leaders.
Managing group/team processes	Includes managing group dynamics, running effective meetings, keeping group focused, establishing team structure, membership, roles and ground rules, creating atmosphere of mutual respect, enhancing communication, building relationships among (not with) team members, empowering group members, fostering democratic/participatory process.
Overcoming resistance to change	Working with leaders and providers (including innovation providers) to overcome resistance to innovation. This activity focuses more on addressing individual attitudes than shared beliefs among members of an organization or sub-groups within an organization.
Collecting data / providing feedback	
Conduct ongoing monitoring of program implementation	Monitoring/tracking/collecting data/information on progress, problems/barriers, enablers (facilitators), fidelity to evidence, performance, and innovation activities and linking implementation to outcomes. Includes quantitative and qualitative data, observations, etc.
Data collection to assess context and <u>baseline</u> performance	Collecting/reviewing quantitative/qualitative diagnostic information/data to understand the local context, baseline performance, determinants of current practice (barriers and enablers (facilitators)).
Providing updates and feedback	Providing updates on implementation, including providing feedback on data, PDSA cycles (CQI), innovation provider activities, facilitator activities, relevant professional or system-level information (e.g., availability of new guidelines, tools, awareness of new administrative/clinical policy, etc.). Includes interpretation of data for stakeholders.

IF Activities	IF Activity Definitions/Examples
Enabling/fostering change	
Fostering organizational change: cultural	Promoting cultural change required for implementation. Includes changes in shared beliefs about care, types of providers' skills and practice scopes (not specific providers, but classes of providers such as primary care providers, social workers, psychiatrists, psychologists, nurses, etc.), efficacy and safety of treatments and practice models. These may be shared beliefs among all members of an organization or members of a sub-group (e.g., mental health providers, nursing, primary care providers, physicians etc.).
Fostering organizational change: structural	Promoting structural change required for implementation (e.g., staffing changes, reporting structure changes, office assignment changes, methods for referring patients, how patients move through the system [e.g., physically get to innovation providers, get from innovation providers to front desk] etc.).
Fostering change, unspecified	Promoting change generally to support implementation, unspecified.
Interceding and liaising with others	Interceding/liaising with leadership (internal or external) and/or relevant programs/departments (internal or external) about the innovation (including regulatory issues, facilitation implementation, other issues) and/or stakeholder needs.
Strategy/policy development	Assisting with strategy and policy development
Helping to define, identify, and fill stakeholder roles	
Describing/clarifying roles and responsibilities	Describing purpose and process of innovation, facilitation, what will occur, establishing/clarifying and allocating roles and responsibilities.
Helping to hire clinical program staff	Assisting with hiring innovation providers, innovation provider replacements or additions; includes helping with writing job descriptions.
Identification/selection of local change agents	Helping identify/select, and/or hire local change agents, e.g., internal facilitators, opinion leaders, champions, and quality improvement (QI) team members.

IF Activities	IF Activity Definitions/Examples
Disseminating innovation or facilitation knowledge	
Attending, presenting at and/or organizing non-local meetings	Organizing, attending and/or presenting at regional/national meetings, councils. advisory boards or other forums (not site visits).
Fostering spread of clinical innovation/facilitation methods	Assisting with spread of clinical innovation and/or facilitation methods beyond facilitation sites.
Planning/preparing for implementation	
Action/implementation planning	Assisting with the development and refinement of Action Plans / Implementation Plans, including formal action items, short-term plans and long-term plans; assisting sites with an implementation checklist.
Adapting program to local context without compromising fidelity	Help to adapt to and create synergy with local context, including local structure, staffing, culture and other initiatives.
Developing shared vision / consensus building	Finding synergy between existing goals and innovation goals; helping stakeholders to “get on the same page”; helping stakeholders to see what is in it for “me and us”; developing “win-win solutions”.
Goal/priority setting	Assisting in setting clear, realistic goals; setting priorities. Includes assisting with the selection of an area for change and developing/refining specific clinical practice questions.
.	
Problem identification and resolution	
Problem identification	Assisting with problem identification, awareness and clarification, including understanding current ways of working and thinking, identifying gaps and barriers in current context.
Problem-solving	Assistance with problem-solving, brainstorming solutions.

**<u>IF Activities</u>**	**<u>IF Activity Definitions/Examples</u>**
Providing administrative/technical support	
Administrative tasks	Set up site visits; scheduling and inviting stakeholders to calls and meetings; organizing meetings; emailing announcements and reminders; setting up monthly innovation provider calls; creating and sending out meeting minutes; preparing and disseminating reports and materials; scheduling/arranging speakers for monthly calls; making arrangements for innovation provider training (e.g., sending innovation providers to other clinics to shadow another innovation provider) (Note that only the *arrangement part* is administrative, not actual *provision* of training); preparing PowerPoint slides. *Does not include organizing regional or national meetings.*
Technical support	Providing technical support (e.g., providing tools and sample materials [e.g., reminder systems, templates, patient materials, etc.]); developing necessary infrastructure to support adoption other than information technology (IT); assisting with conducting literature searches and appraising and summarizing the evidence; working with stakeholders to create documents/evaluation materials. May also include identifying or providing information about other available resources, such as resources available in community. Also working with IT or other stakeholders to ensure that IT systems accurately capture and support innovation activity (e.g., encounters) or anything related to computer support issues including software.
Providing education/information	
Clinical skills education	Providing education/information about <u>clinical content</u> skills/expertise, including provider education, clinical training/supervision/coaching and mentoring, academic detailing (can be ongoing). Also includes counseling, co-counseling, critical reflection, new ways of work, experiential learning, learning from practice. *Does not include presentations at regional and or national meetings.*
Marketing	Promoting or publicizing the innovation and what it does, including provision of evidence for innovation conducted by facilitators, highlighting a need for practice change, emphasizing enhanced patient outcomes as reason for change, and interpreting the research for practical application.
Marketing education	Education about how to conduct marketing to clinic providers, staff or leadership. Includes teaching innovation providers how to market their own skills and services
Organizational change skills education	Providing education/information about change skills/processes, including skills, incentives and resources to implement change and information about organizational systems and change processes, evaluation, how to engage stakeholders in change process, empowering participants.

IF Activities	IF Activity Definitions/Examples
Using interpersonal skills to create a supportive environment	
Providing support	Being generally helpful and available; communicating regularly; being available for questions; providing encouragement; doing things in a warm, encouraging, and empathetic way rather than hypercritical, punishing way; demonstrating “people skills”; using carrots rather than sticks; acknowledging ideas and efforts and celebrating achievements/success; maintaining momentum and enthusiasm; creating an open, supportive, and trusting environment conducive to change; providing ongoing support/reassurance and constructive feedback. May also include self-disclosure (sharing personal insights or experiences), interjecting humor.
Pulling back and letting sites take lead	Pulling back and letting sites take lead in implementation / sustainability
