# Supplementary material for: Getting to Fidelity: Consensus Development Process to Identify Core Activities of Implementation Facilitation
Source: Glob Implement Res Appl. Author manuscript; Available in PMC 2024 May 17. (PMC11100021; doi:10.1007/s43477-024-00119-5)
Supplement: Online Resource 1 [file NIHMS1990339-supplement-Online_Resource_1.pdf]

Online Resource 1. Description of high and low complexity clinical innovation examples provided to expert panel

High Complexity Innovation: Implementing Measurement Based Care for Depression in Integrated Primary Care Mental Health Integration (PCMHI) Settings

Background

Major Depression Disorder (MDD) is a chronic and debilitating disease occurring in up to 12% of men and 25% of women during their lifetime (DSM-IV TR). Multiple studies have shown that symptom monitoring is often inadequate, resulting in clinician failure to detect medication noncompliance, suboptimal pharmacotherapy dosing or augmentation, or failure to add psychotherapy as needed (Simon 1998, Kroenke 2000). Less than 35% of patients receiving care experience symptom remission and these limited responses to treatment are not accompanied by changes in the treatment plan (Trevedi 2004, 2006). Almost a third of patients receiving care for depression do so in the primary care setting and less than half (41.1%) receive adequate care (Kessler 2003).

Recognizing the need to support the provision of mental health care in the primary care setting, integration of mental health care into Veteran Health Administration (VHA) primary care (PC) settings has been the expected standard of care for medical centers and large (greater than 5,000 unique patient population) community based outpatient clinics (CBOCs) since 2008. As part of the Primary Care Mental Health Integration (PCMHI) initiative, VHA mandates implementation of *both* Co-Located Collaborative Care and Care Management. Co-Located Collaborative Care embeds psychiatrists, psychologists and/or social workers in PC teams and addresses mental health disorders, as well as behavioral factors that negatively impact chronic medical conditions (Pomerantz, 2010). Embedded providers staff open-access clinics, with PC providers conducting warm handoffs in real time. Subsequently scheduled or walk-in encounters are often provided concurrently with scheduled PC encounters. In Care Management models, care managers support PC providers by conducting face-to-face or telephone patient encounters and providing patient education/activation, barrier assessment/ resolution, symptom monitoring, medication adherence and side-effects monitoring, and/or self-management goal setting and monitoring.

Practice change

The Patient Health Questionnaire (PHQ-9), a nine-item questionnaire, has been established as a brief diagnostic and severity instrument that can be used to screen for depression as well as measure changes in symptomatology over time to continuously monitor and inform adjustments to treatment as needed (Kroenke, 2002). The PHQ-9 can be self-administered or included in a clinical interview. Documenting and responding to patient's symptoms changes can provide the foundation upon which providers can deliver the patient centered care that VHA strives to achieve. Annually, VA mandated screening for depression using the PHQ-2 and structured assessments using the PHQ-9 is now in place (Yano, 2012). In addition, the PHQ-9 is currently used as a core component of symptom monitoring in PCMHI Care Management. Yet, only 45% of VA PC clinics have incorporated the Care Management component of PCMHI, while 85% report having Co-Located Collaborative Care.

The PHQ-9 is available in an electronic format in the VHA electronic medical record through *The Mental Health Assistant (MHA)* and a patient's changes in the PHQ-9 can be viewed graphically by front line providers within the MHA. Yet, routine administration of the PHQ-9 as an outcome measure in PCMHI co-located collaborative care is rare and the use of the PHQ-9 beyond the annual depression screening is not monitored by VHA. VHA PC and mental health leadership convened an expert panel and charged it to identify PCMHI goals and objectives given the reorganization of VA primary care into Patient-Aligned Care Teams (PACT). One of the six recommendations from this panel is that **"The PCMHI integrated mental health care providers should offer consistent follow-up assessments and monitoring of patient outcomes with appropriate adjustment in treatments…"**. This recommendation is substantially supported by multiple systematic reviews which have found that patient outcome monitoring is a core component of multifaceted interventions that have had the greatest impact in improving depression treatment and outcomes in primary care (Gilbody 2003, Williams 2007, Rubenstein 2009).

Processes integral to implementing measurement-based care for depression in integrated primary care mental health settings

(1) Identify the processes for providing measurement-based care

  (a) Process for identifying PC patients due for their annual PHQ-2/9 screening

  (b) Process for identifying PC patients in need of ongoing PHQ-9 monitoring after screening positive for depression and initiating treatment

(2) Identify a process to administer the PHQ-9 prior to the interaction with the provider

  (a) Need a local procedure for obtaining PHQ-9

  (b) Need mechanism for feeding back this information to health care providers for discussion with patients, to inform adjustments to treatment if needed, and to document adjustments to treatment in response to PHQ-9 outcome monitoring

(3) Document impact of PHQ-9 outcome monitoring on depression treatment adjustment and symptom improvement

Low Complexity Innovation: Improving Patient Safety for Veterans Taking Antipsychotic Medications

Background

Psychotic disorders are prevalent, disabling and costly among Veterans receiving health care services in the VA and elsewhere. While 3.4% of VA service users have a diagnosis of schizophrenia, patients with this serious mental illness (SMI) account for 11.7% of VA health care costs. In FY10, about 242,000 patients with a psychotic disorder diagnosis (e.g., schizophrenia, bipolar disorder) were treated in the VA. Of these, 62.1% received one or more prescriptions for oral antipsychotics and of those treated with antipsychotic medication, 91.2% were prescribed a second-generation antipsychotic (SGA). Unfortunately, treatment with many SGAs is associated with adverse metabolic side effects such as overweight/obesity, diabetes, and dyslipidemia. Failure to properly monitor and manage these side effects can lead to increased risk of mortality due to diabetic ketoacidosis and cardiovascular disease. In addition to these treatment-emergent adverse effects, patients with SMIs such as schizophrenia already have greater prevalence of obesity (42%) and diabetes (13%) than the general population.

A VA Office of the Inspector General (OIG) report found that a substantial proportion of patients prescribed antipsychotics who were overweight did not receive weight management interventions. The OIG report and VA research clearly demonstrate the need for timelier monitoring and management of antipsychotics' metabolic side effects. In response to the OIG report and emerging research findings, VA's Office of Mental Health Services formed the Atypical Antipsychotic Workgroup to develop recommendations focused on improving processes for identifying individuals at risk for adverse antipsychotic side effects and implementing practices to reduce those risks. One key strategy that informs the other strategies recommended by the Workgroup is routine metabolic monitoring of antipsychotic side effects. This recommended strategy is consistent with VA/DoD clinical practice guidelines for psychoses, obesity, diabetes, and dyslipidemia. Specific implementation strategies for supporting related practice changes were not included in the recommendations.

Practice Change

Monitoring of weight or body mass index (BMI), blood pressure (BP), fasting plasma glucose (FPG), and fasting plasma lipids is recommended when a patient is started on a new antipsychotic medication, with follow-up monitoring to include weight/BMI assessment monthly or at each visit; and BP, FPG and lipid profile at 3-4 months and then annually. For the purposes of this exercise, we will focus ONLY on developing an implementation strategy to improve BASELINE monitoring of weight/BMI, BP, FPG and fasting plasma lipids.

Processes integral to improving patient safety for Veterans taking a new antipsychotic

- For patients started on a new antipsychotic prescription…
  - Obtain baseline weight or BMI, fasting plasma glucose and fasting plasma lipids
    - Need a local procedure for obtaining current weight or BMI
    - Need a local procedure for obtaining fasting blood labs (glucose, lipids)
    - Need mechanism for feeding back this information to health care providers (i.e., whether monitoring was completed and results)
